# Research on Bamboo Shoot Bud Development: A Leap from Tissue Heterogeneity to Single-Cell Spatial Atlas

**DOI:** 10.3390/plants15081233

**Published:** 2026-04-16

**Authors:** Ying Li, Xueping Li, Zhimin Gao

**Affiliations:** National State Forestry and Grassland Administration Key Open Laboratory on the Science and Technology of Bamboo and Rattan, Institute of Gene Science and Industrialization for Bamboo and Rattan Resources, International Centre for Bamboo and Rattan, Beijing 100102, China; liying@icbr.ac.cn

**Keywords:** Moso bamboo, shoot bud development, single-cell transcriptomics, spatial transcriptomics, chromatin accessibility, cellular heterogeneity

## Abstract

China has rich bamboo resources, with Moso bamboo (*Phyllostachys edulis*) being the most economically important species. Bamboo shoot bud development directly determines the eating quality of the shoots and the properties of bamboo materials; however, the intrinsic biological characteristics of this process have hindered foundational research. Traditional methods using whole shoot buds or mixed tissues obscure cellular and tissue heterogeneity, limiting our mechanistic understanding. This review synthesizes cytological features, molecular networks, and technical limitations pertaining to Moso bamboo shoot bud development, identifying four key bottlenecks: tissue homogenization masking cellular heterogeneity, loss of spatial positional information impeding analysis of position effects, challenges in single-cell technology application due to sample preparation and data interpretation issues, and unresolved coupling between chromatin accessibility and transcriptional regulation. To address these, we propose a core strategy centered on constructing a single-cell resolution, spatially resolved, multi-omics integrated, and functionally validated framework. Key approaches include developing bamboo-specific single-cell sequencing and spatial transcriptomics, integrating positional information with multi-omics data to identify spatially distinct regulatory targets, standardizing technical pipelines and functional validation platforms, and elucidating epigenetic–transcriptional coupling. Overcoming these bottlenecks will reveal the molecular basis of bamboo’s unique developmental patterns and provide key targets for the genetic improvement of the shoot quality and mechanical properties of bamboo.

## 1. Introduction

China is endowed with abundant bamboo resources, ranking first in the world in terms of species diversity, forest area, and standing stock [1]. Bamboo forests serve key ecological and economic functions, such as water regulation, food supply, carbon sequestration, and income generation, and thus play a crucial role in national development strategies—including ensuring timber security, achieving the “dual carbon” goals, and promoting rural revitalization [2]. However, due to the long-term wild state of bamboo plants, their large genome sizes, inherent genetic barriers, long flowering cycles, and the fact that most bamboo plants die after flowering, basic research on bamboo remains relatively underdeveloped [3,4]. These factors constitute the root causes of many “bottleneck” problems, severely constraining genetic breeding efforts regarding bamboo and profoundly affecting the depth and breadth of technological innovation in the bamboo industry.

**Figure 1:**
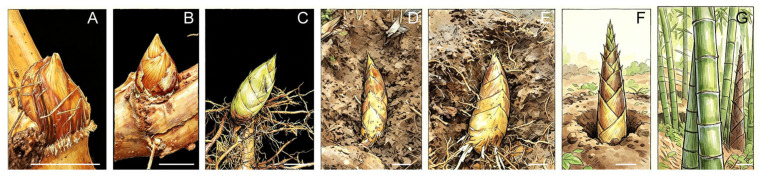
Moso bamboo shoot bud development at different stages. (**A**) Germination stage; (**B**) Early developmental stage; (**C**) Middle developmental stage; (**D**) Late developmental stage; (**E**) Mature stage; (**F**) Spring bamboo shoot with sheaths; (**G**) Culm. Scale bars: (**A**,**B**) 1 cm; (**C**–**E**) 10 cm; (**F**,**G**) 15 cm.

Moso bamboo is an economically important bamboo species in China, with the largest planted area, widest range of applications, and most advanced research and development among bamboo species [5,6]. It belongs to the genus *Phyllostachys* (subfamily Bambusoideae) and is a typical clonal plant, propagating asexually mainly through rhizomes (underground stems) bearing buds as well as through bamboo culms and branches [7,8]. Starting in August, lateral buds on the rhizomes exit dormancy and begin to sprout (Figure 1A), entering a developmental period (Figure 1B–E). These buds generally mature around February of the following year and emerge from the soil (Figure 1F). Although bamboo lacks fascicular cambium in its vascular bundles and does not undergo the seasonal radial growth typical of perennial trees such as poplars, the shoot buds nevertheless undergo significant thickening during underground tissue differentiation and maturation. By the time of shoot bud maturation (i.e., when the winter bamboo shoots have emerged), the basic structure of the future aboveground culm has already formed, and the tissues and organs are well developed. At this stage, shoot diameter, the number of vascular bundles in the shoot wall, the wall-to-cavity ratio, and the number nodes are largely fixed. After emergence, the shoots (i.e., spring bamboo shoots) primarily undergo internode elongation and dry-matter accumulation (Figure 1G) [9,10].

Thus, the developmental process of shoot buds is critical for determining traits related to shoot quality and bamboo culm properties. Systematically elucidating the regulatory mechanisms underlying shoot bud development in Moso bamboo is not only essential for advancing fundamental theories regarding the key traits of shoot quality and culm properties but also significant in terms of applicational value for their improvement, representing an important research direction in bamboo science.

## 2. Search Methods

A literature search was conducted using the electronic databases PubMed, Web of Science, Google Scholar, and China National Knowledge Infrastructure (CNKI). The search terms included combinations of the following keywords: “Plants”, “Bamboo”, “Shoot bud”, “Single-cell RNA sequencing”, “Spatial Transcriptomics”, and “Chromatin accessibility”. Only peer-reviewed articles published in Chinese or English from 1970 to 2026 were considered. Additional relevant studies were identified by manually screening the reference lists of the retrieved articles.

## 3. Morphological and Cytological Basis of Shoot Bud Development in Moso Bamboo

**Figure 2:**
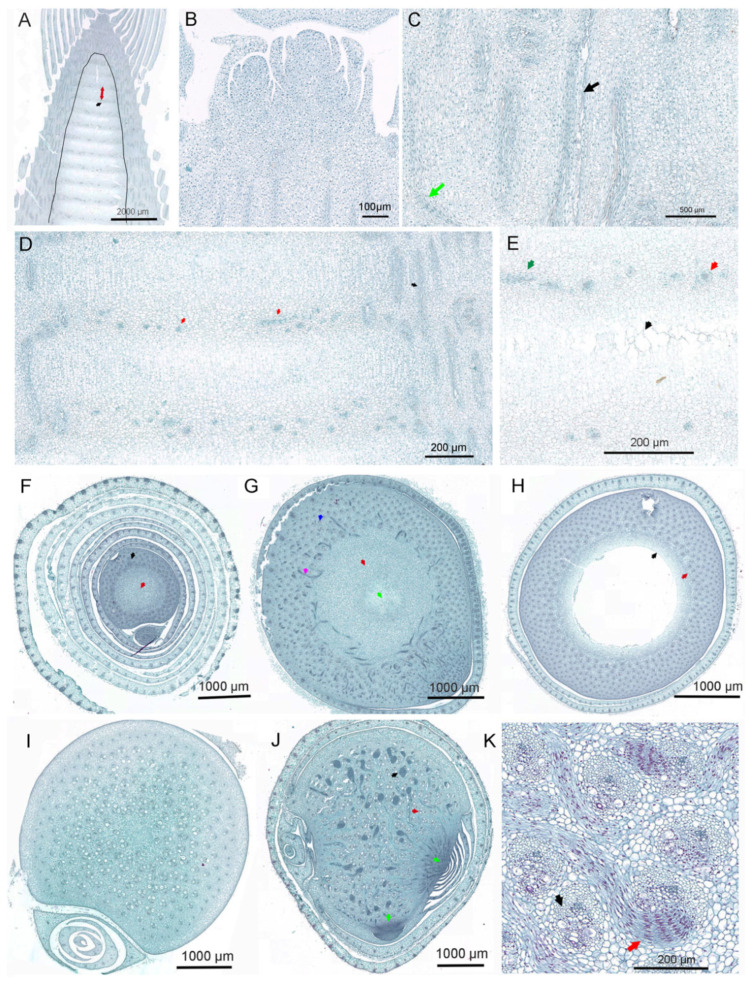
Morphological characteristics of meristematic cells in a developing Moso bamboo shoot bud at the mid-growth stage. (**A**) Longitudinal section of a bamboo shoot bud at the mid-growth stage. The black curved box delineates the pith region (inside) and the shoot wall (outside). Black arrows indicate developing nodes, and the red arrow indicates the developing internode. (**B**–**E**) Higher-magnification views of regions in (**A**). (**B**). Stratified longitudinal vascular bundles at the shoot apex. (**C**) Longitudinal vascular bundles in the shoot wall. Black arrows denote longitudinal vascular bundles adjacent to the pith region; green arrows denote longitudinal vascular bundles curving toward the culm sheath. (**D**) Immature nodes and nodal septa in the pith region. (**E**) Node and nodal septum regions in the pith. Black arrows denote pith cells between two nodal septa that mature and rupture first; red and green arrows denote transverse vascular bundles in the nodal septum, shown in vertical and parallel sections, respectively. (**F**) Cross-section at the node position near the shoot apex. The red arrow denotes the pith region; the black arrow denotes the shoot wall. (**G**) Cross-section at the node position in the mid-shoot region. Green arrows denote inner pith cells; red arrows denote outer pith cells; pink arrows denote transverse vascular bundles; blue arrows denote longitudinal vascular bundles in the shoot wall. (**H**) Cross-section at the internode position in the mid-shoot region. Black and red arrows denote inner and outer pith cells, respectively. (**I**) Cross-section of the basal region of the shoot. (**J**) Cross-section of the basal shoot at the nodal septum region. The green arrow denotes bud primordium; red arrows denote mature longitudinal vascular bundles; black arrows denote transverse vascular bundles. (**K**) Transverse (red arrows) and longitudinal (black arrows) vascular bundles undergoing differentiation and development in the basal region of the shoot. Distinct differences in nuclear chromatin morphology (stained bright red) are evident.

Shoot bud development occurs underground, where the activities of the apical meristem drive the progressive differentiation and maturation of multiple meristems, along with the gradual specification of cell fates. This process is characterized by diverse meristem types, strict spatiotemporal regulation, pronounced cellular heterogeneity, and a highly dynamic chromatin structure (Figure 2).

### 3.1. Characteristics of Tissue Differentiation During Development

Prior to shoot maturation, cells in the tunica and corpus of the apical meristem sequentially differentiate into prophyll primordia, longitudinally and transversely oriented procambial cells, and pith meristematic cells. Subsequently, these meristematic cells give rise to structures such as the shoot wall, pith cavity, nodes, internodes, shoot-borne roots, and prophylls, during which the shoot bud continuously thickens. Following the end of dormancy, cell activity in the apical meristem of the shoot bud significantly increases during the germination stage. In mid-developmental shoots, tissues such as epidermal cells, longitudinally layered procambium, nodal diaphragms, and pith are clearly distinguishable (Figure 2A).

The development of the longitudinally layered procambium in the shoot wall is asynchronous: it initiates in the lower portion and occurs slightly later in the mid-to-upper portion (Figure 2B). Even within the same cross-section, its morphology is not uniform—the procambium near the pith region remains relatively straight, whereas that at the node curves toward the prophyll (Figure 2C). The procambium sequentially differentiates from the inner to outer layers into procambial bundles, which subsequently develop into vascular tissues. From the early stages of development, the pith meristem undergoes pronounced differentiation. Internode cells are gradually arranged in columns, clearly distinguishing them from nodal cells (Figure 2D). As shoot development progresses, the internodal meristematic cells in the pith region adjacent to the shoot wall continue to differentiate, whereas the innermost parenchyma cells of the pith cavity gradually mature and subsequently rupture (Figure 2E), at which point the nodal diaphragm structure begins to take shape.

### 3.2. Spatiotemporal Asynchrony of Development and Cell Fate Determination

Notably, although shoot bud development initiates in the apical meristem, basal development precedes that of the apex (Figure 2F,G). In later developmental stages, a pith ring forms between the pith region and the shoot wall, marking the completion of pith meristem differentiation and development (Figure 2H). The vascular bundles at the base of the shoot bud mature first, and during subsequent stages, lateral root primordia sequentially differentiate at the base (Figure 2I,J). As development proceeds, dense rooting occurs at the base of the culm, and root emergence through the epidermis signals the onset of the maturation stage in *P. edulis* shoot buds. Notably, the structure and morphology of nuclear chromatin undergo pronounced changes during tissue development (Figure 2K). Collectively, these observations indicate that shoot bud development is a continuous process in which the differentiation and maturation of various meristems, although not fully synchronous, are mutually interdependent.

## 4. Current Research Status and Limitations of the Regulatory Mechanisms of Bamboo Shoot Bud Development

Numerous studies have examined bamboo shooting patterns, the morphological structures of shoot buds, metabolite changes, and taxonomy, with key findings focusing on the anatomical structure of various shoot bud tissues [11], the phenological patterns of bud development [12], endogenous hormones during bud differentiation and development [13], and changes in proteins, amino acids, and lipids [14]. These results indicate that during shoot bud development, cell morphology and the structure of chromatin undergo significant changes, accompanied by active metabolism of both biomacromolecules (e.g., proteins and lipids) and small-molecule phytohormones. Moreover, the shooting process influences photosynthesis, nutrient uptake, and utilization in bamboo stands, thereby affecting forest structure and ecological function.

### 4.1. Advances in Transcriptome Research in the Era of High-Throughput Sequencing

In recent years, researchers have employed high-throughput sequencing technologies to investigate the growth and development of bamboo shoot buds. Wei et al. (2017) [15] conducted cytological and transcriptomic analyses of several bamboo species with varying culm wall thicknesses, including Moso bamboo. Their results revealed a reduction in both the number of cell layers and the number of cells per layer in the shoot apical meristem during late developmental stages. In the early developmental stage, genes associated with vascular development—primarily involved in plant hormone signal transduction and cell wall development—were identified in shoot buds. The same team also found that during medullary cavity formation in *Pseudosasa japonica*, genes related to programmed cell death, ethylene and calcium-ion signaling pathways, and protein hydrolysis were differentially expressed [16]. Using wild-type Moso bamboo and its thick-walled variant *P*. *edulis* f. pachyloen as experimental materials, Li et al. (2022) [17] collected shoot buds at five stages (germination, early development, mid-development, late development, and maturity) and performed multi-omics sequencing. They discovered that during shoot bud thickening, miR167, miR160, and miR396, together with genes encoding transcription factors such as PeGRFs and PeARFs, form miRNA–mRNA modules involved in biological pathways including plant hormone signal transduction, carbohydrate metabolism, and microtubule movement. These modules regulate key processes such as cell wall synthesis, cell wall configuration, and lateral root growth in bamboo shoot buds. Furthermore, higher cytokinin levels, or a lower auxin-to-cytokinin ratio, were found to favor shoot bud tissue differentiation.

### 4.2. Core Bottlenecks and Unresolved Mysteries in Existing Research

Currently, several questions regarding shoot bud development remain unresolved. For example, are the vertically arranged procambium within the culm wall and the horizontally arranged procambium at the nodal diaphragms homologous? Why is there a marked inconsistency in the maturation of cells between the inner and outer layers of the lamellar procambium in the shoot wall? Why do internodes above the root-forming internode undergo successive, similar developmental processes? Why does the maturation of medullary meristem cells exhibit an “endarch” pattern? Constrained by the limitations of previous research techniques, studies have often used entire shoot buds, shoots after sheath removal, or partial shoots as materials. The averaged data derived from such mixed tissues inevitably mask the heterogeneity among distinct cell populations. Importantly, miRNAs have been confirmed to be mobile signaling molecules in plants [18,19,20]. Therefore, resolving gene transcriptional heterogeneity at the single-cell spatial level and conducting analyses based on established miRNA–mRNA modules will provide new perspectives and important references for an in-depth elucidation of miRNA regulatory mechanisms. In conclusion, shoot bud development is governed by a complex interactive network involving multiple stages, levels, and factors. Although numerous genes associated with shoot bud development have been identified (and are still being identified), the connections between these genes and the progressive differentiation and maturation of various tissues remain far from clear.

## 5. Cutting-Edge Technology: Application Potential and Challenges Regarding Single-Cell and Spatial Transcriptomics

Plant single-cell transcriptomic technology enables in-depth investigation of cellular heterogeneity and cell state diversity. By tracing developmental lineages across distinct cell types, this approach reveals the heterogeneity of gene expression among different cell types within tissues or organs, identifies key regulatory elements that determine cell fate, and provides important guidance for plant development and future breeding efforts [21].

### 5.1. The Development of Single-Cell Transcriptomics in Plant Research

Single-cell sequencing was first reported in 2009 [22]. Over the past decade, particularly in recent years, single-cell transcriptomic sequencing has undergone explosive advances and seen widespread adoption [23,24]. Microfluidics-based isolation and barcoding of messenger RNA from individual cells have enabled truly high-throughput single-cell sequencing [25], fundamentally transforming our understanding of gene expression at the single-cell level and offering new perspectives on cellular heterogeneity, identity, and function [26,27,28].

Single-cell transcriptomics offers unique advantages in the study of plant development, largely due to the immobility of plant cells and their fixed spatial positions. Furthermore, after embryogenesis, organs such as leaves, roots, and flowers develop from meristems composed of stem cell populations [29]. In recent years, innovations in plant protoplast isolation techniques have facilitated the rapid expansion of single-cell transcriptomic studies across diverse tissue types and species [30]. Single-cell transcriptomic studies in the model plant Arabidopsis thaliana marked the beginning of plant single-cell genomics [31,32,33,34,35]. Subsequently, single-cell-resolution 3D imaging was achieved in the shoot apical meristem, florets, and root apical meristem of rice [36], and single-cell transcriptomic maps of the rice root tip [37] and leaf [38] were successively generated, alongside studies on the developmental trajectories of rice florets and inflorescence meristems [39]. More recently, single-cell transcriptomic atlases have been completed for wheat leaf guard cells [40], wheat spike development [41], maize shoot apex development [42], and cotton fibers [43,44]. For the perennial tree species poplar, single-cell transcriptomic maps of the stem have also been obtained [45,46,47].

### 5.2. Current Status and Limitations of Single-Cell Research in Bamboo Plants

Collectively, these studies demonstrate that single-cell RNA sequencing (scRNA-seq) of plants enables comprehensive characterization of both common and rare cell types and states, facilitates the construction of developmental transcriptomic maps at single-cell resolution, allows tracing of cell lineage developmental processes, supports analysis of dynamic cell-state transitions, permits investigation of intercellular communication, and aids in the dissection of transcription factor regulatory networks. In recent years, single-cell technologies have matured and been widely applied, with studies now extending to bamboo. Researchers have successfully generated single-cell transcriptomic atlases of bamboo roots [48], single-internode stems [49], intercalary meristem [50], and shoot buds [51]. However, owing to technical limitations and the lack of mature genetic transformation systems for bamboo, current studies remain largely confined to cell-type classification and transcriptional characterization, with insufficient functional validation of key regulatory genes. Moreover, a systematic integration of single-cell atlases across different bamboo tissues is lacking, and a comprehensive single-cell expression atlas for bamboo has yet to be established.

### 5.3. Spatially Resolved Transcriptomics: A Key to Deciphering the “Position Effect”

Cell differentiation and cell fate are governed by multiple factors. In addition to cell type, function, and developmental timing, spatial positional information also exerts a significant influence on the regulation of cell fate and the evolution of cell lineages. Halle et al. (1978) [52] provided a comprehensive description of positional effects, suggesting that the state of the meristem is determined by its position within the plant and remains stable during vegetative propagation. Cong (1999) [53] proposed that the differentiation and development of tissues and organs in higher plants are pre-determined in the meristem, where the division of specific cells dictates the position and structure of tissues and organs. Xiong (1980) [54] also noted that tissue differentiation and organ formation in bamboo shoot buds are not entirely dependent on cell origin, as they are also related to cell position.

Although collecting samples at different timepoints, combined with single-cell transcriptome sequencing, allows for the analysis of cell types and gene expression patterns across temporal dimensions, scRNA-seq requires the dissociation of cells from tissues, resulting in a loss of spatial positional information. Consequently, the spatial coordinates of genes within cells cannot be obtained. Therefore, to systematically elucidate the molecular mechanisms underlying developmental processes such as cell division and the progressive differentiation and maturation of multiple tissues in bamboo shoot buds, it is necessary to integrate spatial information to investigate transcriptional heterogeneity, intercellular interactions, and regulation during these processes, thereby revealing the spatial and developmental relationships among cell types [55].

Spatial omics technology based on single-cell RNA sequencing can capture cellular spatial information [56]. This technology enables the visualization of spatial gene expression, allowing for the identification of distinct cell populations while preserving their spatial locations. It thus clarifies the relationships among cell function, phenotypes, and spatial position within the tissue microenvironment, offering new perspectives for studying gene expression and tissue complexity [57]. Although scientists predict that this technology will play a pivotal role in plant science research [58,59,60], its application has so far been limited to a few plants, such as wheat [41], poplar [47] and bamboo [50], due to challenges such as the highly specialized nature of plant tissues. While some progress has been made in these species, widespread adoption has not yet been achieved.

### 5.4. Practical Bottlenecks of the Technology Used in Bamboo Shoot Bud Applications

Therefore, integrating single-cell and spatial transcriptomic technologies through combined data analysis enables the localization of specific transcripts and the identification of the cell subpopulations that produce them. This approach will help elucidate the transcriptional regulatory mechanisms underlying the differentiation and maturation of various tissues in bamboo shoot buds, particularly those with “homeotic” characteristics, such as the procambium, vascular tissue, and nodal diaphragms. Nevertheless, the small size of bamboo shoot buds—especially at early developmental stages—blurs the spatial mapping boundaries between different cell types. Furthermore, given the diversity of cell types and the compactness of tissue structures, precisely controlling tissue permeabilization time to ensure adequate mRNA release from various tissues without causing diffusion to non-target areas remains a major technical bottleneck in applying this technology to bamboo shoot buds.

## 6. Epigenetic Regulation: The Role of Chromatin Accessibility in Shoot Bud Development

In multicellular organisms, despite sharing the same genomic information, morphologically diverse cell nuclei generate distinct transcriptional landscapes, thereby acquiring unique fates and biological functions. This cell-type-specific transcription is closely linked to chromatin accessibility. Chromatin accessibility—also referred to as chromatin openness—involves dynamic changes in higher-order chromatin structures that regulate gene activation, silencing, and transcriptional levels, ultimately determining tissue-specific cell fate [61]. Importantly, the physical accessibility of chromatinized DNA undergoes frequent remodeling in response to environmental changes and during developmental progression [62,63].

### 6.1. Regulatory Mechanisms of Chromatin Accessibility

Chromatin accessibility reflects the extent to which physical contact is permitted between cis-regulatory elements—such as promoters, enhancers, silencers, and insulators—and chromatin DNA [64]. This accessibility arises from the combined effects of nucleosome occupancy, topological configuration, transcription factor binding, and chromatin remodeling complexes [65]. As the core structural units of chromatin, nucleosomes not only provide the structural basis for DNA winding [66,67,68] but also play critical roles in regulating gene expression [69]. In transcriptionally active regions, i.e., open chromatin regions, nucleosome arrays are relatively loosely arranged [70], and the intervening DNA sequences frequently interact with regulatory proteins, including transcription factors, RNA polymerases, and structural proteins.

Owing to their DNA sequence specificity, transcription factors play a central organizing role in the remodeling of chromatin accessibility. Thurman et al. (2012) [71] demonstrated that although accessible chromatin constitutes only 2–3% of the human genome sequence, it encompasses more than 90% of transcription-factor-binding regions. Furthermore, post-translational histone modifications influence the functional state of nucleosomes and modulate chromatin accessibility through multiple mechanisms. These include steric hindrance of transcription factor binding by nucleosomes and regulation of nucleosome affinity for active chromatin-remodeling factors [72,73]. For instance, Xie et al. (2016) [74] reported that histone acetylation neutralizes the positive charge of lysine residues, thereby weakening histone–DNA interactions and relaxing chromatin structure, which facilitates transcription factor or transcriptional regulator binding and promotes gene transcription. Qin et al. (2023) [75] discovered that environmental metabolites can regulate gene transcription through chromatin modifications.

### 6.2. Research Progress on Chromatin Accessibility in Plants and Research Gaps in Shoot Bud Studies

In recent years, advances in plant single-nucleus isolation techniques have revealed extensive single-cell accessibility diversity across various plant tissue types. Using rice root tip cells, Feng et al. (2022) [76] employed a single-nucleus assay for transposase-accessible chromatin sequencing (snATAC-seq) [77] to uncover cell-type-specific accessibility differences and identified transcription factor sets associated with accessible chromatin regions. Studies on rice radicle [78] and wheat embryo [79] development have integrated single-cell RNA sequencing, chromatin accessibility assays, and histone modification profiling to dissect cell type specification processes, transcriptomic landscapes, and marker genes, thereby reconstructing continuous developmental trajectories of specific cell lineages and elucidating the regulatory networks underlying cell fate determination. Collectively, these findings indicate that dynamic changes in chromatin accessibility landscapes broadly reflect their regulatory capacity and serve as key determinants of chromatin organization and function.

Pronounced heterogeneity in nuclear chromatin structure and morphology has been observed across tissues and cell populations during bamboo shoot bud development using optical microscopy, accompanied by spatiotemporally specific changes along the developmental trajectory. These preliminary findings suggest that chromatin accessibility remodeling occurs during bamboo shoot bud development and that histone modifications may regulate transcription factor activity by modulating histone–DNA interactions, thereby playing critical roles in establishing and maintaining cell identity in bamboo shoot buds. However, the influence of chromatin accessibility on gene expression regulation during bamboo shoot bud development remains unclear, necessitating an integrative analysis of these multilayered epigenetic features. Elucidating the sequence determinants of accessible chromatin, predicting potential transcription factor binding, and uncovering the impact of post-translational histone modifications on nucleosome occupancy will help us understand the molecular mechanisms underlying spatiotemporally specific transcriptional regulation in bamboo shoot buds.

## 7. Key Challenges in Current Research

In summary, bamboo shoot bud development is a continuous process characterized by the progressive differentiation and maturation of multiple meristems, driven by the activity of the apical meristem. However, previous studies on the primary thickening growth of bamboo shoot buds exhibit several major limitations. First, although temporally resolved transcriptomic studies have been conducted using samples from different developmental stages, technical constraints have largely necessitated the use of whole or partial shoot tissues. This approach masks inter-tissue and inter-cellular heterogeneity, making it difficult to resolve cell types, their origins, and transcriptional heterogeneity. Second, although numerous genes and regulatory factors involved in bamboo shoot bud development have been identified, the lack of spatial position information hinders interpretation of cellular “positional effects,” thereby limiting its value for guiding precise breeding of important traits in Moso bamboo. Third, the application of single-cell technology to bamboo shoot buds currently faces challenges such as a weak foundational omics reference base, a lack of spatial information, difficulties hampering cell dissociation techniques, insufficient resolution of developmental dynamics, inconsistent nomenclature systems, and a lack of functional validation platforms. These issues collectively limit the “credibility” of cell annotation and the “depth of biological insight” that can be achieved. Fourth, plant differentiation and development are governed by the interplay of functional genes, regulatory factors, and epigenetic modifications. Chromatin accessibility has been demonstrated to be closely linked to plant differentiation and development. Although notable changes in nuclear chromatin structure have been observed during shoot development, the relationship between chromatin accessibility and transcriptional regulation in bamboo remains unclear.

## 8. Future Directions and Strategic Priorities

To address these challenges, future efforts should focus on establishing single-cell and spatial transcriptomic technologies as a new paradigm. With the rapid advancement of single-cell sequencing (scRNA-seq/scATAC-seq) and spatial transcriptomics, bamboo research is poised to transition from traditional “tissue homogenization” to analyses with “spatially resolved, single-cell resolution.” A critical prerequisite for this is the development of an efficient protoplast isolation protocol for Moso bamboo, which would directly address the current technical bottleneck. To this end, we propose constructing a bamboo single-cell reference atlas using representative species such as Moso bamboo, covering distinct developmental stages to systematically define cell types and their molecular characteristics. This should be integrated with spatial transcriptomics (e.g., 10× Visium) and in situ validation techniques such as single-molecule fluorescence in situ hybridization (smFISH) or immunohistochemistry. Furthermore, given the challenges posed by the abundant polysaccharides, polyphenols, and cellulose in bamboo shoot buds, it is essential to develop bamboo-specific cell dissociation and nuclear extraction protocols to improve cell viability and data quality.

Building on these technological advances, integrating spatial information with multi-omics data will enhance the precision of targeted breeding. Elucidating positional effects will elevate gene function studies from the “cell type” level to the “spatial microenvironment” level, providing spatially resolved molecular targets for important agronomic traits such as internode elongation, vascular bundle distribution, and shoot wall thickening. We recommend constructing “gene–cell–space” association networks by integrating single-cell data with spatial transcriptomics to identify key regulators specifically expressed in particular spatial regions (e.g., meristematic zones, vascular bundle sheaths, and pith). A prerequisite for applying spatial transcriptomics to complex samples such as bamboo shoot buds—which contain multiple types of densely distributed meristems—is to determine the optimal tissue permeabilization time. Subsequently, developing a spatially resolved gene-editing validation system using bamboo callus transformation or transient expression systems with tissue-specific promoters, along with a “spatial QTL” mapping strategy, will add a new dimension to marker-assisted breeding.

Finally, to ensure reproducibility and scalability, it is critical to strengthen the fundamental aspects of single-cell technologies and promote standardization and the development of functional validation platforms. A bamboo single-cell database with a unified nomenclature system should be established to facilitate cross-study comparisons. To resolve developmental dynamics, RNA velocity and pseudotime analysis should be employed, complemented by genetic lineage tracing systems (e.g., CRISPR-Cas9-mediated systems). Furthermore, efficient functional validation platforms are urgently needed, including for transient transformation of bamboo protoplasts, stable transformation of calli, and virus-induced gene silencing (VIGS). Concurrently, deeper investigation into the coupling between chromatin accessibility and transcriptional regulation will help elucidate epigenetic regulatory networks. We recommend performing single-cell multi-omics joint analysis (e.g., 10× Multiome) to construct regulatory networks comprising cis-regulatory elements, transcription factors, and target genes. Key cis-regulatory elements should be identified via ATAC-seq and validated through conservation analysis and transgenic assays. Finally, integrating ChIP-seq with hormone treatment experiments will reveal how epigenetic modifications mediate spatially specific responses to hormone signaling during shoot development.

## 9. Conclusions

**Figure 3:**
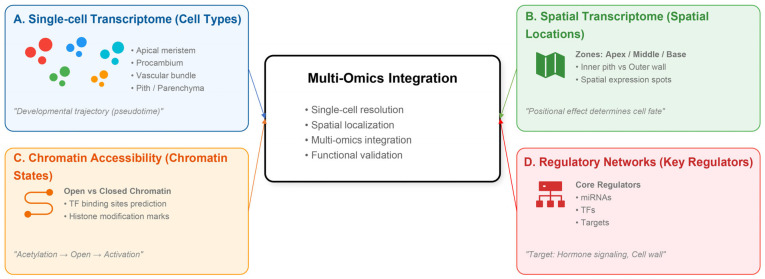
Multi-omics research framework for Moso bamboo shoot bud development. The framework systematically integrates single-cell transcriptomics ((**A**), cell types), spatial transcriptomics ((**B**), spatial locations), chromatin accessibility ((**C**), chromatin states), and gene regulatory networks ((**D**), key regulators). The different colored dots in subfigure (**A**) represent distinct cell types.

The primary thickening growth of bamboo shoot buds is a highly ordered, spatiotemporally specific developmental process. Future research should strategically adopt a core approach centered on “single-cell resolution, spatial localization, multi-omics integration, and closed-loop functional validation” to systematically address current technical bottlenecks and biological questions (Figure 3). Overcoming these challenges will not only drive fundamental breakthroughs in the developmental biology of bamboo but also provide robust molecular targets and theoretical support for precision breeding of economically important species such as Moso bamboo, thereby bridging the gap between basic plant science and practical agronomic improvement.

## Data Availability

No new data were created or analyzed in this study.

## Abbreviations

The following abbreviations are used in this manuscript:

CNKI	China National Knowledge Infrastructure
scRNA-seq	Single-cell RNA sequencing
QTL	Quantitative trait locus
VIGS	Virus-induced gene silencing
scATAC-seq	Single-cell assay for transposase-accessible chromatin using sequencing
smFISH	Single-molecule fluorescence in situ hybridization
